# Maternal Pre-pregnancy Body Mass Index Categories and Infant Birth Outcomes: A Population-Based Study of 9 Million Mother–Infant Pairs

**DOI:** 10.3389/fnut.2022.789833

**Published:** 2022-02-17

**Authors:** Xin'nan Zong, Huan Wang, Liu Yang, Yajun Guo, Min Zhao, Costan G. Magnussen, Bo Xi

**Affiliations:** ^1^Department of Epidemiology, School of Public Health, Qilu Hospital, Cheeloo College of Medicine, Shandong University, Jinan, China; ^2^Department of Growth and Development, Capital Institute of Pediatrics, Beijing, China; ^3^Department of Epidemiology, School of Public Health and Tropical Medicine, Tulane University, New Orleans, LA, United States; ^4^Department of Toxicology and Nutrition, School of Public Health, Cheeloo College of Medicine, Shandong University, Jinan, China; ^5^Menzies Institute for Medical Research, University of Tasmania, Hobart, TAS, Australia; ^6^Research Centre of Applied and Preventive Cardiovascular Medicine, University of Turku, Turku, Finland; ^7^Centre for Population Health Research, University of Turku, Turku University Hospital, Turku, Finland

**Keywords:** pre-pregnancy, body mass index, obesity, preterm birth, low birthweight, macrosomia, small for gestational age, large for gestational age

## Abstract

**Background and Aims:**

Infant adverse birth outcomes have been suggested to contribute to neonatal morbidity and mortality and may cause long-term health consequences. Although evidence suggests maternal prepregnancy body mass index (BMI) categories associate with some birth outcomes, there is no consensus on these associations. We aimed to examine the associations of maternal prepregnancy BMI categories with a wide range of adverse birth outcomes.

**Methods:**

Data were from a population-based retrospective cohort study of 9,282,486 eligible mother–infant pairs in the U.S. between 2016 and 2018. Maternal prepregnancy BMI was classified as: underweight (<18.5 kg/m^2^); normal weight (18.5–24.9 kg/m^2^); overweight (25.0–29.9 kg/m^2^); obesity grade 1 (30–34.9 kg/m^2^); obesity grade 2 (35.0–39.9 kg/m^2^); and obesity grade 3 (≥40 kg/m^2^). A total of six birth outcomes of the newborn included preterm birth, low birthweight, macrosomia, small for gestational age (SGA), large for gestational age (LGA), and low Apgar score (5-min score <7).

**Results:**

Maternal prepregnancy overweight and obesity increased the likelihood of infant preterm birth, with odds ratios (ORs) (95% CIs) of 1.04 (1.04–1.05) for overweight, 1.18 (1.17–1.19) for obesity grade 1, 1.31 (1.29–1.32) for obesity grade 2, and 1.47 (1.45–1.48) for obesity grade 3, and also for prepregnancy underweight (OR = 1.32, 95% CI = 1.30–1.34) after adjusting for all potential covariates. Prepregnancy overweight and obesity were associated with higher odds of macrosomia, with ORs (95% CIs) of 1.53 (1.52–1.54) for overweight, 1.92 (1.90–1.93) for obesity grade 1, 2.33 (2.31–2.35) for obesity grade 2, and 2.87 (2.84–2.90) for obesity grade 3. Prepregnancy overweight and obesity was associated with higher odds of LGA, with ORs (95% CIs) of 1.58 (1.57–1.59) for overweight, 2.05 (2.03–2.06) for obesity grade 1, 2.54 (2.52–2.56) for obesity grade 2, and 3.17 (3.14–3.21) for obesity grade 3. Prepregnancy overweight and obesity were also associated with higher odds of low Apgar score, with ORs (95% CIs) of 1.12 (1.11–1.14) for overweight, 1.21 (1.19–1.23) for obesity grade 1, 1.34 (1.31–1.36) for obesity grade 2, and 1.55 (1.51–1.58) for obesity grade 3.

**Conclusion:**

Our findings suggest maintaining or obtaining a healthy body weight for prepregnancy women could substantially reduce the likelihood of important infant adverse birth outcomes.

## Introduction

Prepregnancy well-being of women of reproductive age and their male partners is the basis of healthy pregnancy that contributes to healthy growth and development of the offspring *in utero*. Some adverse birth outcomes such as preterm birth, low birthweight, or macrosomia are important clinical and public health concerns as they increase the incidence of death of the newborns and might lead to long-term health consequences (1–3). Maternal prepregnancy malnutrition is also one of the most common clinical phenomena that may affect birth outcomes of the newborns. It is estimated that global obesity prevalence will surpass 21% and severe obesity will surpass 9% in women by 2025 (4). Among US women, an increasing trend in obesity was found, with the prevalence from 31.5 in 2011 to 2012 to 56.9% in 2017 to 2018 (5). Thus, it is very necessary to explore the associations between maternal prepregnancy body mass index (BMI) and a wide range of adverse birth outcomes.

Maternal prepregnancy overweight and obesity have been associated with several adverse birth outcomes, including preterm birth, macrosomia, and large for gestational (LGA), and prepregnancy underweight is associated with low birthweight and small for gestational age (SGA) (2, 6). However, there is no consensus on these associations. A meta-analysis of 11 studies including 452,991 participants showed no significant association between prepregnancy obesity and preterm birth (odds ratio [OR] = 1.21, 95% CI: 0.95–1.53) and a weak association of prepregnancy underweight with preterm birth (OR = 1.13, 1.01–1.27) (7). Another meta-analysis of 60 studies including 1,392,799 participants reported that prepregnancy obesity had a positive association with low birthweight (OR = 1.24, 1.09–1.41) and a weak association with preterm birth (OR = 1.05, 1.01–1.09) (8). A cohort study among 12,029 pregnant women suggested that prepregnancy obesity increased the risk of LGA and even SGA (9). In addition, although a meta-analysis of 11 studies including 2,586,265 participants suggested maternal overweight and obesity were associated with low Apgar score (10), authors of a subsequent population-based cohort (*N* = ~2,000) concluded there was no difference in the odds of low Apgar score among offspring of women with normal weight, overweight and obesity before pregnancy (11). Thus, the inconsistent associations of maternal prepregnancy BMI categories with a wide range of adverse birth outcomes require confirmation.

In this study, we examined the associations of maternal prepregnancy BMI categories (from underweight to obesity grade 3) with a wide range of infant adverse birth outcomes (i.e., preterm birth, low birthweight, macrosomia, SGA, LGA, and low Apgar score) in a population-based national study of 9 million mother–infant pairs.

## Materials and Methods

### Data Source

We used national birth certificate data from the U.S. National Vital Statistics System (NVSS), which is a retrospective, population-based cohort study that includes information on a wide range of maternal and infant demographics and health characteristics for all births occurring in 50 states and the District of Columbia in the U.S. Birth certificate data from each registration area are received by the Vital Statistics Cooperative Program, the National Center for Health Statistics of the Centers for Disease Control and Prevention. The NVSS data collection methodology, quality control, and vital statistics are publicly available on the CDC website (https://www.cdc.gov/nchs/nvss/births.htm).

**Figure 1:**
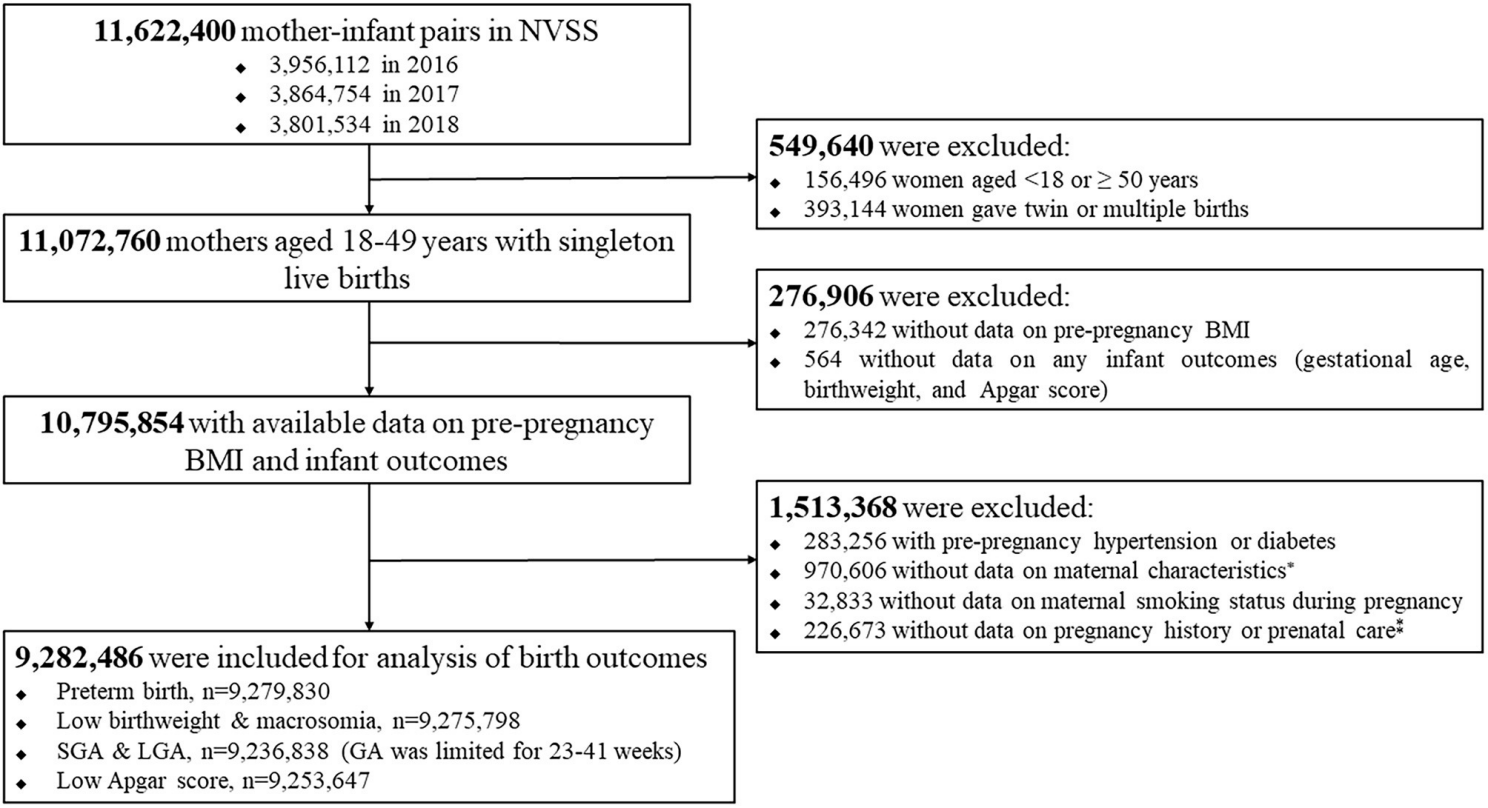
Flow chart of inclusion/exclusion of the study population. *maternal age at delivery, race/ethnicity, education level, marital status; ^‡^The number of live births, and the total number of prenatal care visits.

In this study, we used 2016–2018 NVSS data because all the US states and the District of Columbia had completely implemented the 2003 version of the Standard Certificate of Live Birth to collect birth information since 2016. We initially extracted all live births of 11,622,400 mother–infant pairs between 2016 and 2018 in the U.S. with 3,956,112 in 2016, 3,864,754 in 2017, and 3,801,534 in 2018. We excluded 549,640 because of age of women <18 or ≥ 50 years, or twin or multiple births, 276,906 because of missing data on maternal prepregnancy BMI, or any infant outcomes (gestational age, birthweight, and 5-min Apgar score), and 1,513,368 because o women with prepregnancy hypertension or diabetes, or missing data on maternal characteristics, maternal smoking status during pregnancy, or pregnancy history or prenatal care. Finally, a total of 9,282,486 eligible mother–infant pairs were included for this analysis. Figure 1 provides a flow chart of inclusion/exclusion of mother–infant participants from the 2016 to 2018 birth certificate data in NVSS.

### Data Collection

Maternal age at delivery was calculated from the date of birth of the mother. Maternal prepregnancy BMI was calculated as prepregnancy weight in kilograms divided by the square of height in meters. Maternal height and weight were self-reported by the women, and weight was maternal weight immediately before the woman became pregnant with the child included in this study. Maternal race/ethnicity was divided into Hispanic, non-Hispanic white, non-Hispanic black, and others. Maternal education levels were collected as the highest level of education at the time of delivery. Marital status was reported as “yes” or “no.” Smoking status during pregnancy was collected as “yes” or “no” during pregnancy. Eclampsia, gestational hypertension, and diabetes, separately, were diagnosed during pregnancy as “yes” and “no.” Live-birth order indicated what number the present birth represents; for example, if a baby is born to a mother who has had two previous live births, this baby will have a live-birth order of the third. A total number of prenatal care visits for this pregnancy was also collected. The infant sex was categorized as male and female. Gestational age was calculated based on the obstetric estimate of gestation at delivery. Infant birth weight was reported in grams, and if the weight in grams was not available, weight in pounds or ounces was converted to grams. Apgar score indicated a systematic measure for evaluating the physical condition of an infant at specific intervals at birth, and a 5-min score was recorded from 0 to 10. Additional information on these variables can be obtained from the User Guides to the 2016–2018 Natality Public Use Files (12–14).

### Definitions of Maternal Prepregnancy BMI Categories and Related Covariates

Maternal prepregnancy BMI was classified as: underweight (<18.5 kg/m^2^); normal weight (18.5–24.9 kg/m^2^); overweight (25.0–29.9 kg/m^2^); obesity grade 1 (30–34.9 kg/m^2^); obesity grade 2 (35.0–39.9 kg/m^2^); and obesity grade 3 (≥ 40 kg/m^2^). We categorized related covariates as follows: maternal age at delivery as <30 years, and ≥ 30 years, maternal race/ethnicity as Hispanic, non-Hispanic white, non-Hispanic black, and other, maternal education level as less than high school, high school, and more than high school, marital status as married and unmarried, smoking status during pregnancy as yes and no, live-birth order as 1, 2, and ≥ 3, infant sex as male and female, total number of prenatal care visits as 0, 1–4, 5–9, and ≥ 10, eclampsia as yes and no, gestational hypertension as yes and no, and gestational diabetes as yes and no.

### Definitions of Birth Outcomes

We included six adverse birth outcomes: preterm birth, low birthweight, macrosomia, SGA, LGA, and low Apgar score. Preterm birth was defined as gestational age at delivery <37 weeks. Low birthweight was defined as birth weight <2,500 g, and macrosomia as birth weight ≥ 4,000 g. SGA and LGA were defined as birth weight below the 10th percentile values (for SGA), and above the 90th percentile values (for LGA) by gestational age and sex according to the U.S. new intrauterine growth curves (15). We defined low Apgar score as a 5-min score less than 7 that indicated an infant in the intermediate or less physical condition.

### Statistical Analysis

Population characteristics were presented using median (interquartile range, IQR) for continuous variables and *n* (%) for categorical variables. Binary or multinomial logistic regression models were used to calculate ORs with 95% CIs of adverse birth outcomes (i.e., preterm birth, low birthweight, macrosomia, SGA, LGA, and low Apgar score) according to maternal prepregnancy underweight, overweight, obesity grade 1, obesity grade 2, and obesity grade 3 relative to the reference group of normal weight. To address the impact of potential confounders, we performed three logistic regression models: Model 1 was unadjusted model; Model 2 was the simpler model adjusted for two demographic factors including maternal age at delivery and race/ethnicity; Model 3 was the model adjusted for more potential confounders to show the more reliable results, including adjustment for maternal age at delivery, race/ethnicity, education levels, marital status, smoking status during pregnancy, live-birth order, infant sex, gestational age (for low birthweight, macrosomia, or low Apgar score only), and a total number of prenatal care visits. We considered the included confounders based on the available variables from the original NVSS data and also from previous similar studies (16, 17). Subgroup analyses were performed by maternal race/ethnicity, maternal age at delivery, and infant birth year. To assess the stability of our findings, two sensitivity analyses were performed by excluding women with cesarean section, and by excluding those with eclampsia, gestational hypertension, or diabetes. We also assessed the dose–response relationship between prepregnancy BMI (as a continuous variable) and infant adverse birth outcomes using restricted cubic spline (RCS) logistic regression models with adjustment for all potential covariates, with three knots of the 5, 50,^,^ and 95th percentiles of the distribution of the continuous prepregnancy BMI (18). RCS_Reg macro was employed for the RCS analysis under SAS 9.4 software. Data cleaning and all analyses were performed by SAS 9.4 (SAS Institute Inc., Cary, North Carolina). A two-sided *P* < 0.05 was considered statistically significant.

## Results

### Characteristics of the Study Population

Table 1 presents characteristics of the study population by maternal prepregnancy BMI category. Among 9,282,486 pregnant women, prepregnancy BMI was divided into underweight (311,445, 3.4%), normal weight (4,067,646, 43.8%), overweight (2,451,775, 26.4%), obesity grade 1 (1,351,922, 14.6%), obesity grade 2 (652,581, 7.0%), and obesity grade 3 (447,117, 4.8%). Overall maternal age at delivery was 29 (IQR: 25–33) years, with 54.9% for <30 years and 45.1% for ≥ 30 years. Overall race/ethnicity consisted of Hispanic (21.5%), non-Hispanic white (55.1%), non-Hispanic black (14.5%), and others (8.9%). Compared with women with prepregnancy normal weight, those with prepregnancy underweight or obesity tended to have lower education levels, be unmarried, and smoke cigarettes during pregnancy.

**Table 1:** Characteristics of the study population by maternal prepregnancy BMI categories.

**Characteristics**	**Total**	**Underweight**	**Normal weight**	**Overweight**	**Obesity grade 1**	**Obesity grade 2**	**Obesity grade 3**
***N***	9,282,486	311,445	4,067,646	2,451,775	1,351,922	652,581	447,117
Pre-pregnancy BMI, kg/m^2^, median (IQR)	25.4(22.1–30.2)	17.7(17–18.1)	22.1(20.7–23.5)	27.1(25.8–28.3)	32.0(30.9–33.3)	37.0(35.9–38.3)	43.4(41.5–46.6)
**Maternal age at delivery, years**							
Median (IQR)	29(25–33)	26(22–31)	29(25–33)	29(25–33)	29(25–33)	28(25–33)	28(25–33)
**Age category, years, n (%)**							
<30	5,100,272(54.9)	212,603(68.3)	2,198,628(54.1)	1,311,719(53.5)	750,054(55.5)	371,385(56.9)	255,883(57.2)
≥30	4,182,214(45.1)	98,842(31.7)	1,869,018(45.9)	1,140,056(46.5)	601,868(44.5)	281,196(43.1)	191,234(42.8)
**Race/ethnicity, n (%)**							
Hispanic	1,999,552(21.5)	50,761(16.3)	761,083(18.7)	606,876(24.8)	344,672(25.5)	148,853(22.8)	87,307(19.5)
Non-Hispanic white	5,112,870(55.1)	168,520(54.1)	2,415,292(59.4)	1,281,733(52.3)	677,208(50.1)	338,348(51.9)	231,769(51.8)
Non-Hispanic black	1,341,257(14.5)	42,231(13.6)	450,686(11.1)	366,026(14.9)	244,148(18.1)	130,976(20.1)	107,190(24.0)
Other	828,807(8.9)	49,933(16.0)	440,585(10.8)	197,140(8.0)	85,894(6.4)	34,404(5.3)	20,851(4.7)
**Maternal education level, n (%)**							
Less than high school	1,125,417(12.1)	47,532(15.3)	438,582(10.8)	320,222(13.1)	184,185(13.6)	82,119(12.6)	52,777(11.8)
High school	2,384,797(25.7)	96,472(31.0)	932,159(22.9)	624,394(25.5)	387,920(28.7)	199,104(30.5)	144,748(32.4)
More than high school	5,772,272(62.2)	167,441(53.8)	2,696,905(66.3)	1507,159(61.5)	779,817(57.7)	371,358(56.9)	249,592(55.8)
**Marital status, n (%)**							
Married	5,673,853(61.1)	166,028(53.3)	2,653,552(65.2)	1,497,432(61.1)	766,320(56.7)	357,360(54.8)	233,161(52.1)
Unmarried	3,608,633(38.9)	145,417(46.7)	1,414,094(34.8)	954,343(38.9)	585,602(43.3)	295,221(45.2)	213,956(47.9)
**Smoking status during pregnancy, n (%)**							
Yes	680,731(7.3)	39,866(12.8)	277,359(6.8)	164,100(6.7)	103,922(7.7)	55,305(8.5)	40,179(9.0)
No	8,601,755(92.7)	271,579(87.2)	3,790,287(93.2)	2,287,675(93.3)	1,248,000(92.3)	597,276(91.5)	406,938(91.0)
**Live birth order, n (%)**							
0	3,512,701(37.8)	143,684(46.1)	168,8065(41.5)	878,229(35.8)	444,064(32.9)	212,726(32.6)	145,933(32.6)
1	3,020,379(32.5)	96,382(31.0)	1,327,603(32.6)	800,879(32.7)	437,795(32.4)	212,006(32.5)	145,714(32.6)
2	1,598,304(17.2)	43,724(14.0)	640,440(15.7)	442,392(18.0)	259,916(19.2)	125,439(19.2)	86,393(19.3)
≥3	1,151,102(12.4)	27,655(8.9)	411,538(10.1)	330,275(13.5)	210,147(15.5)	102,410(15.7)	69,077(15.5)
**Infant sex, n (%)**							
Male	4,748,518(51.2)	158,795(51.0)	2,081,128(51.2)	1,255,650(51.2)	691,613(51.2)	333,228(51.1)	228,104(51.0)
Female	4,533,968(48.8)	152,650(49.0)	1,986,518(48.8)	1,196,125(48.8)	660,309(48.8)	319,353(48.9)	219,013(49.0)
**Total number of prenatal care visits, n (%)**							
0	144,306(1.6)	7,027(2.3)	65,013(1.6)	38,035(1.6)	19,931(1.5)	8,725(1.3)	5,575(1.3)
1–4	343,212(3.7)	15,243(4.9)	146,893(3.6)	91,002(3.7)	50,747(3.8)	23,422(3.6)	15,905(3.6)
5–9	1,927,755(20.8)	73,168(23.5)	841,602(20.7)	511,705(20.9)	281,822(20.9)	131,127(20.1)	88,331(19.8)
≥10	6,867,213(74.0)	216,007(69.4)	3,014,138(74.1)	1,811,033(73.9)	999,422(73.9)	489,307(75.0)	337,306(75.4)

*BMI, body mass index; IQR, interquartile range*.

### Associations of Maternal Prepregnancy BMI Categories With Infant Birth Outcomes

#### Preterm Birth

In the fully adjusted model, maternal prepregnancy underweight was associated with higher odds of preterm birth (OR = 1.32, 95% CI = 1.30–1.34); prepregnancy overweight and obesity also increased the odds of preterm birth, with ORs (95% CIs) of 1.04 (1.04–1.05) for overweight, 1.18 (1.17–1.19) for obesity grade 1, 1.31 (1.29–1.32) for obesity grade 2, and 1.47 (1.45–1.48) for obesity grade 3 (Table 2).

**Table 2:** Odds ratios and 95% CIs of infant birth outcomes according to maternal prepregnancy BMI categories.

**Birth outcomes**	**Underweight**	**Normal weight**	**Overweight**	**Obesity grade 1**	**Obesity grade 2**	**Obesity grade 3**
**Preterm birth (<37 weeks)**						
n (%)	29,679(9.5)	278,909(6.9)	177,198(7.2)	109,659(8.1)	57,470(8.8)	43,863(9.8)
Model 1	1.43(1.41–1.45)	1.00	1.06(1.05–1.07)	1.20(1.19–1.21)	1.31(1.30–1.32)	1.48(1.46–1.49)
*P*-value	<0.0001		<0.0001	<0.0001	<0.0001	<0.0001
Model 2	1.42(1.41–1.44)	1.00	1.03(1.02–1.03)	1.15(1.14–1.16)	1.25(1.24–1.26)	1.39(1.37–1.40)
*P-*value	<0.0001		<0.0001	<0.0001	<0.0001	<0.0001
Model 3	1.32(1.30–1.34)	1.00	1.04(1.04–1.05)	1.18(1.17–1.19)	1.31(1.29–1.32)	1.47(1.45–1.48)
*P*-value	<0.0001		<0.0001	<0.0001	<0.0001	<0.0001
**Low birthweight (<2,500 g)**						
n (%)	34,251(11.0)	247,138(6.1)	136,544(5.6)	79,574(5.9)	39,689(6.1)	28,970(6.5)
Model 1	1.84(1.82–1.86)	1.00	0.94(0.93–0.95)	1.01(1.00–1.02)	1.06(1.05–1.08)	1.16(1.14–1.17)
*P*-value	<0.0001		<0.0001	0.0127	<0.0001	<0.0001
Model 2	1.79(1.76–1.81)	1.00	0.90(0.89–0.91)	0.94(0.93–0.95)	0.98(0.97–0.99)	1.03(1.02–1.04)
*P-*value	<0.0001		<0.0001	<0.0001	<0.0001	<0.0001
Model 3	1.64(1.61–1.66)	1.00	0.79(0.78–0.80)	0.72(0.71–0.72)	0.65(0.64–0.66)	0.59(0.58–0.60)
*P*-value	<0.0001		<0.0001	<0.0001	<0.0001	<0.0001
**Macrosomia (≥4,000 g)**						
n (%)	8,718(2.8)	260,257(6.4)	218,481(8.9)	137,928(10.2)	75,351(11.6)	57,103(12.8)
Model 1	0.45(0.44–0.46)	1.00	1.43(1.42–1.43)	1.66(1.65–1.68)	1.92(1.90–1.93)	2.16(2.14–2.19)
*P*-value	<0.0001		<0.0001	<0.0001	<0.0001	<0.0001
Model 2	0.48(0.47–0.49)	1.00	1.49(1.48–1.49)	1.78(1.77–1.79)	2.06(2.04–2.07)	2.35(2.33–2.38)
*P-*value	<0.0001		<0.0001	<0.0001	<0.0001	<0.0001
Model 3	0.54(0.52–0.55)	1.00	1.53(1.52–1.54)	1.92(1.90–1.93)	2.33(2.31–2.35)	2.87(2.84–2.90)
*P*-value	<0.0001		<0.0001	<0.0001	<0.0001	<0.0001
**SGA (< P**_**10**_ **)** ^**†**^						
n (%)	34,334(11.1)	254,306(6.3)	124,159(5.1)	65,399(4.9)	30,419(4.7)	19,844(4.5)
Model 1	1.81(1.79–1.83)	1.00	0.82(0.82–0.83)	0.80(0.79–0.81)	0.79(0.78–0.80)	0.77(0.76–0.78)
*P*-value	<0.0001		<0.0001	<0.0001	<0.0001	<0.0001
Model 2	1.70(1.68–1.72)	1.00	0.80(0.79–0.80)	0.75(0.75–0.76)	0.73(0.72–0.74)	0.69(0.68–0.70)
*P-*value	<0.0001		<0.0001	<0.0001	<0.0001	<0.0001
Model 3	1.56(1.54–1.58)	1.00	0.81(0.80–0.82)	0.77(0.76–0.77)	0.74(0.73–0.75)	0.69(0.68–0.70)
*P*-value	<0.0001		<0.0001	<0.0001	<0.0001	<0.0001
**LGA (>P**_**90**_ **)** ^**†**^						
n (%)	7,177(2.3)	199,779(4.9)	183,242(7.5)	125,343(9.3)	72,877(11.2)	59,500(13.4)
Model 1	0.48(0.47–0.49)	1.00	1.55(1.54–1.56)	1.95(1.94–1.97)	2.40(2.38–2.42)	2.93(2.90–2.96)
*P*-value	<0.0001		<0.0001	<0.0001	<0.0001	<0.0001
Model 2	0.52(0.51–0.53)	1.00	1.60(1.59–1.61)	2.07(2.05–2.08)	2.56(2.54–2.58)	3.18(3.14–3.21)
*P-*value	<0.0001		<0.0001	<0.0001	<0.0001	<0.0001
Model 3	0.54(0.53–0.55)	1.00	1.58(1.57–1.59)	2.05(2.03–2.06)	2.54(2.52–2.56)	3.17(3.14–3.21)
*P*-value	<0.0001		<0.0001	<0.0001	<0.0001	<0.0001
**Low Apgar score (<7 scores at 5 min)**						
n (%)	5,134(1.7)	62,133(1.5)	42,052(1.7)	26,493(2.0)	14,892(2.3)	12,442(2.8)
Model 1	1.08(1.05–1.11)	1.00	1.12(1.11–1.14)	1.29(1.27–1.31)	1.51(1.48–1.53)	1.84(1.81–1.88)
*P*-value	<0.0001		<0.0001	<0.0001	<0.0001	<0.0001
Model 2	1.05(1.02–1.08)	1.00	1.12(1.10–1.13)	1.26(1.24–1.28)	1.44(1.42–1.47)	1.72(1.68–1.75)
*P-*value	0.0018		<0.0001	<0.0001	<0.0001	<0.0001
Model 3	0.88(0.86–0.91)	1.00	1.12(1.11–1.14)	1.21(1.19–1.23)	1.34(1.31–1.36)	1.55(1.51–1.58)
*P*-value	<0.0001		<0.0001	<0.0001	<0.0001	<0.0001

*BMI, body mass index; LGA, large for gestational age; SGA, small for gestational age*.

† *SGA and LGA were defined as birth weight below the 10th percentile (for SGA), and above the 90th percentile (for LGA) by gestational age and sex according to the U.S. new intrauterine growth curves*.

*Maternal pre-pregnancy normal weight was the reference group*.

*Model 1: Unadjusted model with only BMI as an explanatory variable*.

*Model 2: Adjusted for maternal age at delivery and race/ethnicity*.

*Model 3: Adjusted for maternal age at delivery, race/ethnicity, education levels, marital status, smoking status during pregnancy, live birth order, infant sex, gestational age (for low birthweight, macrosomia, or low Apgar score only), and the total number of prenatal care visits*.

#### Low Birthweight and Macrosomia

In the fully adjusted model, maternal prepregnancy underweight was associated with increased odds of low birthweight, with OR (95% CI) of 1.64 (1.61–1.66), and prepregnancy overweight, and obesity were also associated with higher odds of macrosomia, with ORs (95% CIs) of 1.53 (1.52–1.54) for overweight, 1.92 (1.90–1.93) for obesity grade 1, 2.33 (2.31–2.35) for obesity grade 2, and 2.87 (2.84–2.90) for obesity grade 3 (Table 2).

#### Small for Gestational Age and Large for Gestational Age

In the fully adjusted model, maternal prepregnancy underweight was associated with higher odds of SGA, with OR (95% CI) of 1.56 (1.54–1.58), and prepregnancy overweight and obesity were associated with higher odds of LGA, with ORs (95% CIs) of 1.58 (1.57–1.59) for overweight, 2.05 (2.03–2.06) for obesity grade 1, 2.54 (2.52–2.56) for obesity grade 2, and 3.17 (3.14–3.21) for obesity grade 3 (Table 2).

#### Low Apgar Score

In the fully adjusted model, maternal prepregnancy overweight and obesity were associated with higher odds of low Apgar score, with ORs (95% CIs) of 1.12 (1.11–1.14) for overweight, 1.21 (1.19–1.23) for obesity grade 1, 1.34 (1.31–1.36) for obesity grade 2, and 1.55 (1.51–1.58) for obesity grade 3 (Table 2).

### Subgroup and Sensitivity Analyses of Maternal Prepregnancy BMI Categories With Infant Birth Outcomes

Subgroup analyses by maternal race/ethnicity, maternal age at delivery, and infant birth year showed largely similar results to those shown for the main analyses (Supplementary Table S1).

Two sensitivity analyses (exclusion of women with cesarean section, exclusion of those with eclampsia, gestational hypertension or diabetes in models) confirmed the consistency of our findings (Supplementary Table S2).

### Dose–Response Relationships of Maternal Pre-pregnancy BMI With Infant Birth Outcomes

**Figure 2:**
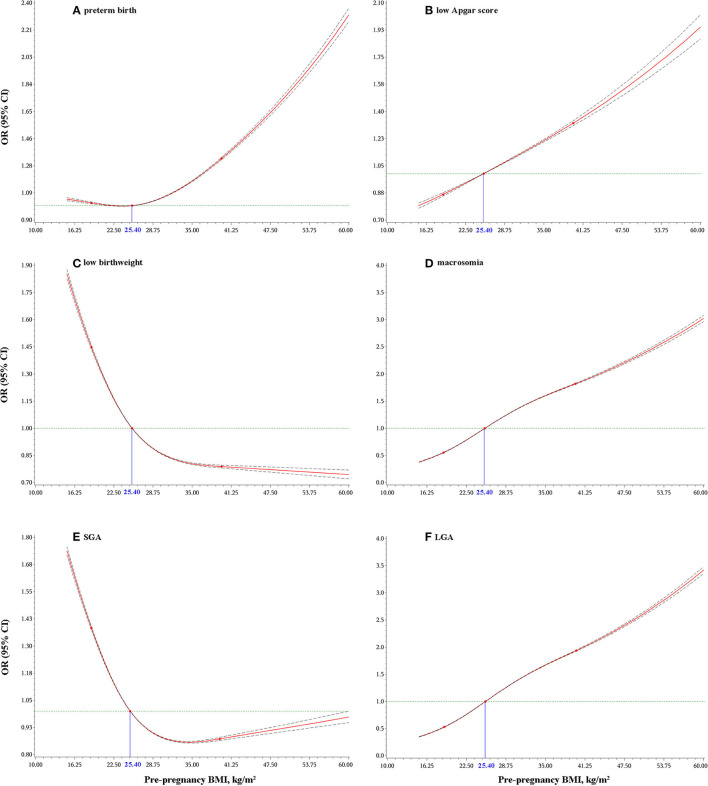
Dose-response relationship of maternal pre-pregnancy BMI with: preterm birth **(A)**; low Apgar score **(B)**; low birthweight **(C)**; macrosomia **(D)**; small for gestational age (SGA) **(E)**; and large for gestational age (LGA) **(F)**. Odds ratios (ORs) and 95% confidence intervals (CIs) were calculated after adjusting for maternal age at delivery, race/ethnicity, education levels, marital status, smoking status during pregnancy, live-birth order, infant sex, gestational age (for low birthweight, macrosomia, or low Apgar score only), and total number of prenatal care visits.

As shown in Figure 2, either a higher or lower maternal prepregnancy BMI increased the odds of preterm birth; a higher maternal prepregnancy BMI was associated with higher odds of macrosomia, LGA and low Apgar score, and a lower maternal prepregnancy BMI was associated with higher odds of low birthweight and SGA. Of note, women with a prepregnancy normal BMI range of 18.5 to 25.0 kg/m^2^ in this observed population was also associated with higher odds of low birthweight and SGA.

## Discussion

Our study examined associations of maternal prepregnancy BMI categories with a wide range of adverse birth outcomes, showing that both prepregnancy overweight/obesity and underweight increased the likelihood of preterm birth; whereas prepregnancy overweight and obesity were associated with an increased likelihood of macrosomia, LGA and low Apgar score, and prepregnancy underweight with higher odds of low birthweight and SGA. However, our data did not lend support to prepregnancy overweight and obesity associating with higher odds of low birthweight and SGA. We also observed the odds of all six outcomes tended to increase as the degree of prepregnancy BMI increased above normal weight. Our findings emphasize the role of a healthy body weight for women of reproductive age, before conception, might play in mitigating the likelihood of infant adverse birth outcomes.

Our findings showed that both maternal prepregnancy underweight and overweight/obesity increased the odds of preterm birth, with the J-shaped relationships of prepregnancy BMI categories with preterm birth, which was independent of maternal age at delivery, race/ethnicity and infant birth year. However, previous studies on the association between maternal prepregnancy weight status and preterm birth has been equivocal (6, 7, 16, 19–21). For example, a meta-analysis including 11 studies (*N* = 452,991) from 6 developing countries showed no significant association between prepregnancy overweight or obesity and preterm birth, and the author's observed high between-study heterogeneity (7). In addition, a meta-analysis including 21 studies (*N* = 678,104) from China showed no significant association between prepregnancy underweight and preterm birth (21). The dissimilar conclusions may be because of the differences in selection of participants, sample size, race/ethnicity, maternal age at delivery, definition of weight categories, and other characteristics of the study populations.

We found that maternal prepregnancy overweight and obesity only increased the odds of macrosomia and LGA rather than low birthweight and SGA, which was consistent with the conclusions of some meta-analyses (8, 22). However, some studies reported that women with prepregnancy obesity had an increased likelihood of both macrosomia and low birthweight (8, 23) or both LGA and SGA (9). Inconsistencies in observed results might be because of the participant selection, statistical power, characteristics of the study populations, and adjustment for different covariates. For example, the association between prepregnancy obesity and low birthweight was substantially reduced and no longer significant after adjustment for gestational age and other maternal factors in a prospective cohort study (24). Based on the largest sample size examined to date, and the use of national birth certificate data, our dose–response analysis suggested that prepregnancy BMI at the lower extreme increased the odds of low birthweight and SGA, and at the upper extreme increased the odds of macrosomia and LGA. Moreover, we also found that women with a prepregnancy normal BMI range of 18.5–25.0 kg/m^2^ in this observed population had higher odds of low birthweight and SGA, indicating that a prepregnancy BMI within this range is not devoid of risk, but this needs further investigation and confirmation—perhaps considering additional factors (e.g., contribution of father or diet quality of pregnant mothers) that might confound or act as an effect modifier of the association.

Our finding showed that there were significant associations between maternal prepregnancy overweight and obesity and low Apgar score. This finding is in accord with a meta-analysis that considered data from 11 studies (*N* = 2,586,265 participants) where maternal overweight and obesity were associated with higher odds of low Apgar scores (10). However, data from some individual cohorts have shown no significant or a weak association between prepregnancy obesity and low Apgar score (11, 25, 26). In addition to the lower statistical power of individual studies compared with meta-analysis, varying measurement of, and adjustment for, potential confounders may also explain these inconsistent findings.

Maternal prepregnancy BMI categories at both upper and lower extremes may affect infant adverse birth outcomes in several ways. First, prepregnancy overweight and obesity usually cause metabolic abnormalities during pregnancy [such as gestational hypertension and diabetes (6, 7)] which may lead to placental abnormalities (27–31), and ultimately affect adverse birth outcomes. Second, excessive or poor maternal periconceptional weight status may increase the risk of abnormal growth and development of the offspring through epigenetic imprinting or methylation (32–34). Third, prepregnancy overweight and obesity may cause the imbalance of maternal intestinal microbiota (35, 36) that may impose an adverse effect on birth outcomes (37).

Our study has several strengths. First, we used the largest sample size with a total of more than 9 million mother–infant pairs from national birth certificate data collected in 50 states and the District of Columbia of the U.S. between 2016 and 2018. Second, we examined a wide range of adverse birth outcomes including preterm birth, low birthweight, macrosomia, SGA, LGA, and low Apgar score. Third, we performed dose–response relationship analysis to assess the stability of our findings by excluding women with cesarean section, and those with eclampsia, gestational hypertension, or diabetes. Fourth, we had data on many potential confounders that allowed us to adjust on the basis of them. However, our study also has limitations. First, maternal prepregnancy BMI was calculated based on self-reported weight and height before pregnancy, but the accurate representation of objective BMI (calculated using measured weight and height) among U.S. women of reproductive age has been shown (38). Second, we did not analyze the association of gestational weight gain with adverse infant birth outcomes in this study since previous studies showed that gestational weight gain presented a weaker association than prepregnancy BMI (39).

## Conclusion

Based on a very large US cohort, we examined the associations between maternal prepregnancy BMI and a number of birth adverse outcomes and performed the dose–response analysis. We found that maternal prepregnancy overweight and obesity associated with the higher odds of preterm birth, macrosomia, LGA, and low Apgar score; maternal prepregnancy underweight is associates with higher odds of preterm birth, low birthweight, and SGA. In consideration of the increasing prevalence of obesity among women of reproductive age worldwide, our findings highlight early health education and implementation of healthy weight management among women planning a pregnancy could substantially reduce the health burden posed by adverse infant birth outcomes.

## Data Availability Statement

The original contributions presented in the study are included in the article/Supplementary Material, further inquiries can be directed to the corresponding author.

## Ethics Statement

Ethical review and approval was not required for the study on human participants in accordance with the local legislation and institutional requirements. The patients/participants provided their written informed consent to participate in this study.

## Author Contributions

BX conceived and designed the study and supervised the study. HW and LY conducted all the analyses. XZ, CM, and BX wrote the manuscript. All the authors carried out the study and interpreted the data.

## Funding

This work was supported by Innovation Team of Climbing Program of Shandong University, and Youth Team of Humanistic and Social Science of Shandong University (20820IFYT1902).

## Conflict of Interest

The authors declare that the research was conducted in the absence of any commercial or financial relationships that could be construed as a potential conflict of interest.

## Publisher's Note

All claims expressed in this article are solely those of the authors and do not necessarily represent those of their affiliated organizations, or those of the publisher, the editors and the reviewers. Any product that may be evaluated in this article, or claim that may be made by its manufacturer, is not guaranteed or endorsed by the publisher.
