# Supplementary material for: Maternal Pre-pregnancy Body Mass Index Categories and Infant Birth Outcomes: A Population-Based Study of 9 Million Mother–Infant Pairs
Source: Front Nutr. 2022 Feb 17;9:789833. doi: 10.3389/fnut.2022.789833 (PMC8891137; doi:10.3389/fnut.2022.789833)
Supplement: Supplementary file 1 [file Table_1.DOC]

Table S1. Odds ratios and 95% confidence intervals of infant birth outcomes according to maternal pre-pregnancy BMI categories stratified by race/ethnicity, maternal age at delivery and infant birth year

	Underweight		Normal weight		Overweight		Obesity grade 1		Obesity grade 2		Obesity grade 3	
	OR (95 % CI)	P value		Ref.		OR (95 % CI)	P value		OR (95 % CI)	P value		OR (95 % CI)	P value		OR (95 % CI)	P value	
Race/ethnicity																	
Hispanic																	
Preterm birth	1.30(1.26-1.34)	<0.0001		1.00		1.06(1.04-1.07)	<0.0001		1.19(1.17-1.21)	<0.0001		1.32(1.29-1.35)	<0.0001		1.48(1.45-1.52)	<0.0001	
Low birthweight	1.67(1.60-1.73)	<0.0001		1.00		0.81(0.79-0.82)	<0.0001		0.73(0.71-0.75)	<0.0001		0.64(0.62-0.67)	<0.0001		0.60(0.58-0.63)	<0.0001	
Macrosomia	0.54(0.51-0.58)	<0.0001		1.00		1.49(1.46-1.51)	<0.0001		1.93(1.90-1.96)	<0.0001		2.42(2.38-2.47)	<0.0001		3.03(2.96-3.11)	<0.0001	
SGA	1.61(1.56-1.66)	<0.0001		1.00		0.80(0.79-0.81)	<0.0001		0.75(0.74-0.77)	<0.0001		0.73(0.71-0.75)	<0.0001		0.70(0.67-0.72)	<0.0001	
LGA	0.57(0.53-0.61)	<0.0001		1.00		1.51(1.48-1.53)	<0.0001		2.06(2.03-2.10)	<0.0001		2.65(2.60-2.70)	<0.0001		3.37(3.29-3.45)	<0.0001	
Low Apgar score	1.00(0.92-1.08)	0.9941		1.00		1.07(1.03-1.10)	<0.0001		1.11(1.07-1.15)	<0.0001		1.20(1.14-1.26)	<0.0001		1.40(1.33-1.49)	<0.0001	
Non-Hispanic white																	
Preterm birth	1.39(1.36-1.41)	<0.0001		1.00		1.06(1.05-1.07)	<0.0001		1.22(1.21-1.24)	<0.0001		1.38(1.37-1.40)	<0.0001		1.62(1.59-1.64)	<0.0001	
Low birthweight	1.70(1.66-1.74)	<0.0001		1.00		0.78(0.76-0.79)	<0.0001		0.73(0.71-0.74)	<0.0001		0.65(0.64-0.67)	<0.0001		0.59(0.57-0.60)	<0.0001	
Macrosomia	0.55(0.53-0.56)	<0.0001		1.00		1.53(1.52-1.54)	<0.0001		1.87(1.85-1.89)	<0.0001		2.26(2.23-2.28)	<0.0001		2.80(2.76-2.84)	<0.0001	
SGA	1.62(1.59-1.65)	<0.0001		1.00		0.80(0.79-0.81)	<0.0001		0.79(0.78-0.80)	<0.0001		0.77(0.76-0.79)	<0.0001		0.72(0.70-0.74)	<0.0001	
LGA	0.55(0.53-0.56)	<0.0001		1.00		1.59(1.58-1.60)	<0.0001		1.99(1.97-2.01)	<0.0001		2.45(2.42-2.48)	<0.0001		3.08(3.04-3.12)	<0.0001	
Low Apgar score	0.86(0.83-0.90)	<0.0001		1.00		1.14(1.12-1.16)	<0.0001		1.25(1.23-1.28)	<0.0001		1.40(1.36-1.43)	<0.0001		1.61(1.56-1.66)	<0.0001	
Non-Hispanic black																	
Preterm birth	1.33(1.29-1.37)	<0.0001		1.00		0.94(0.93-0.95)	<0.0001		1.01(1.00-1.03)	0.1578		1.11(1.09-1.13)	<0.0001		1.17(1.15-1.20)	<0.0001	
Low birthweight	1.50(1.45-1.55)	<0.0001		1.00		0.79(0.78-0.81)	<0.0001		0.71(0.69-0.72)	<0.0001		0.66(0.64-0.68)	<0.0001		0.60(0.59-0.62)	<0.0001	
Macrosomia	0.60(0.55-0.65)	<0.0001		1.00		1.46(1.43-1.49)	<0.0001		1.79(1.74-1.83)	<0.0001		2.06(2.00-2.12)	<0.0001		2.55(2.47-2.63)	<0.0001	
SGA	1.40(1.36-1.44)	<0.0001		1.00		0.82(0.81-0.83)	<0.0001		0.77(0.75-0.78)	<0.0001		0.74(0.72-0.75)	<0.0001		0.68(0.67-0.70)	<0.0001	
LGA	0.66(0.60-0.71)	<0.0001		1.00		1.50(1.46-1.53)	<0.0001		1.88(1.83-1.93)	<0.0001		2.24(2.17-2.30)	<0.0001		2.80(2.71-2.88)	<0.0001	
Low Apgar score	0.91(0.85-0.97)	0.0049		1.00		1.08(1.05-1.11)	<0.0001		1.12(1.08-1.16)	<0.0001		1.21(1.16-1.25)	<0.0001		1.39(1.34-1.45)	<0.0001	
Other																	
Preterm birth	1.05(1.02-1.09)	0.0051		1.00		1.11(1.09-1.14)	<0.0001		1.24(1.21-1.28)	<0.0001		1.25(1.20-1.30)	<0.0001		1.40(1.33-1.47)	<0.0001	
Low birthweight	1.47(1.41-1.53)	<0.0001		1.00		0.87(0.85-0.90)	<0.0001		0.72(0.69-0.75)	<0.0001		0.64(0.61-0.69)	<0.0001		0.52(0.48-0.57)	<0.0001	
Macrosomia	0.48(0.45-0.51)	<0.0001		1.00		1.65(1.62-1.69)	<0.0001		2.40(2.33-2.47)	<0.0001		3.21(3.09-3.33)	<0.0001		3.83(3.66-4.00)	<0.0001	
SGA	1.44(1.40-1.48)	<0.0001		1.00		0.88(0.86-0.90)	<0.0001		0.77(0.75-0.80)	<0.0001		0.69(0.65-0.72)	<0.0001		0.59(0.55-0.63)	<0.0001	
LGA	0.45(0.42-0.49)	<0.0001		1.00		1.74(1.70-1.79)	<0.0001		2.56(2.49-2.64)	<0.0001		3.48(3.35-3.61)	<0.0001		4.19(4.01-4.37)	<0.0001	
Low Apgar score	0.84(0.77-0.92)	0.0001		1.00		1.18(1.13-1.24)	<0.0001		1.33(1.26-1.41)	<0.0001		1.54(1.42-1.66)	<0.0001		1.82(1.66-1.99)	<0.0001	
Maternal age																	
<30 years																	
Preterm birth	1.35(1.33-1.37)	<0.0001		1.00		0.98(0.97-0.99)	<0.0001		1.07(1.06-1.08)	<0.0001		1.17(1.15-1.18)	<0.0001		1.30(1.28-1.32)	<0.0001	
Low birthweight	1.65(1.62-1.68)	<0.0001		1.00		0.78(0.77-0.79)	<0.0001		0.72(0.71-0.73)	<0.0001		0.65(0.64-0.66)	<0.0001		0.60(0.59-0.62)	<0.0001	
Macrosomia	0.54(0.52-0.55)	<0.0001		1.00		1.51(1.50-1.53)	<0.0001		1.87(1.85-1.89)	<0.0001		2.24(2.21-2.27)	<0.0001		2.76(2.72-2.80)	<0.0001	
SGA	1.55(1.53-1.58)	<0.0001		1.00		0.81(0.80-0.81)	<0.0001		0.77(0.76-0.78)	<0.0001		0.74(0.73-0.75)	<0.0001		0.70(0.69-0.71)	<0.0001	
LGA	0.55(0.53-0.56)	<0.0001		1.00		1.56(1.55-1.58)	<0.0001		2.00(1.98-2.03)	<0.0001		2.46(2.43-2.49)	<0.0001		3.10(3.05-3.14)	<0.0001	
Low Apgar score	0.88(0.85-0.91)	<0.0001		1.00		1.10(1.08-1.12)	<0.0001		1.18(1.16-1.20)	<0.0001		1.30(1.27-1.33)	<0.0001		1.46(1.42-1.50)	<0.0001	
≥30 years																	
Preterm birth	1.20(1.17-1.23)	<0.0001		1.00		1.13(1.12-1.14)	<0.0001		1.32(1.30-1.33)	<0.0001		1.48(1.46-1.50)	<0.0001		1.68(1.65-1.71)	<0.0001	
Low birthweight	1.61(1.57-1.66)	<0.0001		1.00		0.81(0.80-0.82)	<0.0001		0.71(0.70-0.72)	<0.0001		0.64(0.63-0.66)	<0.0001		0.57(0.55-0.58)	<0.0001	
Macrosomia	0.53(0.51-0.55)	<0.0001		1.00		1.53(1.52-1.55)	<0.0001		1.94(1.93-1.96)	<0.0001		2.41(2.38-2.44)	<0.0001		2.97(2.93-3.02)	<0.0001	
SGA	1.59(1.55-1.62)	<0.0001		1.00		0.82(0.81-0.83)	<0.0001		0.77(0.75-0.78)	<0.0001		0.74(0.73-0.76)	<0.0001		0.68(0.66-0.69)	<0.0001	
LGA	0.53(0.51-0.55)	<0.0001		1.00		1.59(1.58-1.60)	<0.0001		2.07(2.04-2.09)	<0.0001		2.60(2.57-2.64)	<0.0001		3.23(3.18-3.28)	<0.0001	
Low Apgar score	0.88(0.83-0.93)	<0.0001		1.00		1.15(1.12-1.17)	<0.0001		1.25(1.22-1.28)	<0.0001		1.38(1.34-1.42)	<0.0001		1.65(1.60-1.71)	<0.0001	
Infant birth year																	
2016																	
Preterm birth	1.32(1.29-1.35)	<0.0001		1.00		1.04(1.03-1.05)	<0.0001		1.17(1.15-1.18)	<0.0001		1.27(1.25-1.29)	<0.0001		1.44(1.41-1.47)	<0.0001	
Low birthweight	1.60(1.56-1.64)	<0.0001		1.00		0.80(0.79-0.81)	<0.0001		0.72(0.71-0.73)	<0.0001		0.66(0.65-0.68)	<0.0001		0.60(0.58-0.62)	<0.0001	
Macrosomia	0.53(0.51-0.55)	<0.0001		1.00		1.54(1.53-1.56)	<0.0001		1.95(1.93-1.98)	<0.0001		2.38(2.34-2.41)	<0.0001		2.91(2.86-2.96)	<0.0001	
SGA	1.54(1.51-1.57)	<0.0001		1.00		0.82(0.81-0.83)	<0.0001		0.78(0.77-0.79)	<0.0001		0.75(0.74-0.77)	<0.0001		0.70(0.68-0.72)	<0.0001	
LGA	0.54(0.52-0.57)	<0.0001		1.00		1.59(1.58-1.61)	<0.0001		2.09(2.06-2.11)	<0.0001		2.59(2.55-2.63)	<0.0001		3.26(3.21-3.31)	<0.0001	
Low Apgar score	0.85(0.81-0.89)	<0.0001		1.00		1.12(1.10-1.15)	<0.0001		1.20(1.17-1.23)	<0.0001		1.30(1.26-1.34)	<0.0001		1.53(1.48-1.59)	<0.0001	
2017																	
Preterm birth	1.32(1.29-1.35)	<0.0001		1.00		1.04(1.03-1.05)	<0.0001		1.18(1.16-1.19)	<0.0001		1.32(1.30-1.34)	<0.0001		1.46(1.43-1.48)	<0.0001	
Low birthweight	1.63(1.59-1.68)	<0.0001		1.00		0.78(0.77-0.80)	<0.0001		0.71(0.69-0.72)	<0.0001		0.64(0.62-0.66)	<0.0001		0.58(0.57-0.60)	<0.0001	
Macrosomia	0.55(0.52-0.57)	<0.0001		1.00		1.53(1.51-1.55)	<0.0001		1.91(1.88-1.93)	<0.0001		2.29(2.25-2.33)	<0.0001		2.87(2.82-2.92)	<0.0001	
SGA	1.56(1.53-1.60)	<0.0001		1.00		0.80(0.79-0.81)	<0.0001		0.76(0.75-0.77)	<0.0001		0.74(0.72-0.76)	<0.0001		0.69(0.67-0.71)	<0.0001	
LGA	0.55(0.53-0.57)	<0.0001		1.00		1.58(1.56-1.60)	<0.0001		2.03(2.00-2.06)	<0.0001		2.52(2.48-2.56)	<0.0001		3.13(3.08-3.19)	<0.0001	
Low Apgar score	0.91(0.86-0.96)	<0.0001		1.00		1.13(1.10-1.15)	<0.0001		1.22(1.19-1.26)	<0.0001		1.35(1.31-1.40)	<0.0001		1.54(1.48-1.59)	<0.0001	
2018																	
Preterm birth	1.32(1.29-1.35)	<0.0001		1.00		1.05(1.04-1.06)	<0.0001		1.19(1.17-1.20)	<0.0001		1.33(1.30-1.35)	<0.0001		1.50(1.47-1.53)	<0.0001	
Low birthweight	1.69(1.65-1.74)	<0.0001		1.00		0.79(0.78-0.80)	<0.0001		0.72(0.70-0.73)	<0.0001		0.64(0.62-0.66)	<0.0001		0.58(0.56-0.60)	<0.0001	
Macrosomia	0.53(0.51-0.55)	<0.0001		1.00		1.52(1.50-1.54)	<0.0001		1.89(1.87-1.91)	<0.0001		2.31(2.27-2.35)	<0.0001		2.83(2.78-2.88)	<0.0001	
SGA	1.58(1.54-1.61)	<0.0001		1.00		0.81(0.80-0.82)	<0.0001		0.76(0.75-0.78)	<0.0001		0.73(0.71-0.74)	<0.0001		0.69(0.67-0.70)	<0.0001	
LGA	0.53(0.51-0.55)	<0.0001		1.00		1.57(1.56-1.59)	<0.0001		2.02(1.99-2.05)	<0.0001		2.51(2.47-2.55)	<0.0001		3.13(3.07-3.18)	<0.0001	
Low Apgar score	0.90(0.85-0.95)	0.0002		1.00		1.12(1.10-1.15)	<0.0001		1.22(1.18-1.25)	<0.0001		1.36(1.31-1.40)	<0.0001		1.57(1.51-1.63)	<0.0001	
BMI, body mass index; LGA, large for gestational age; SGA, small for gestational age; OR, odds ratio; CI, confidence interval; Ref, reference
ORs (95% CIs) were adjusted for maternal age at delivery, race/ethnicity, education levels, marital status, smoking status during pregnancy, live-birth order, infant sex, gestational age (for low birthweight, macrosomia, or low Apgar score only), and total number of prenatal care visits; maternal pre-pregnancy normal weight was the reference group.
 
Table S2. Sensitivity analyses of the associations between maternal pre-pregnancy BMI categories and infant birth outcomes

	Underweight		Normal weight		Overweight		Obesity grade 1		Obesity grade 2		Obesity grade 3	
	OR (95 % CI)	P value		Ref.		OR (95 % CI)	P value		OR (95 % CI)	P value		OR (95 % CI)	P value		OR (95 % CI)	P value	
Excluding women with caesarean section																	
Preterm birth	1.33(1.31-1.35)	<0.0001		1.00		1.03(1.02-1.04)	<0.0001		1.15(1.13-1.16)	<0.0001		1.26(1.24-1.28)	<0.0001		1.43(1.41-1.45)	<0.0001	
Low birthweight	1.67(1.64-1.70)	<0.0001		1.00		0.77(0.77-0.78)	<0.0001		0.70(0.69-0.71)	<0.0001		0.63(0.61-0.64)	<0.0001		0.59(0.57-0.60)	<0.0001	
Macrosomia	0.55(0.54-0.56)	<0.0001		1.00		1.46(1.45-1.47)	<0.0001		1.74(1.72-1.76)	<0.0001		2.01(1.98-2.03)	<0.0001		2.25(2.21-2.28)	<0.0001	
SGA	1.58(1.56-1.60)	<0.0001		1.00		0.79(0.78-0.80)	<0.0001		0.74(0.73-0.74)	<0.0001		0.70(0.69-0.71)	<0.0001		0.65(0.63-0.66)	<0.0001	
LGA	0.54(0.53-0.56)	<0.0001		1.00		1.51(1.50-1.53)	<0.0001		1.86(1.84-1.88)	<0.0001		2.22(2.19-2.25)	<0.0001		2.54(2.50-2.58)	<0.0001	
Low Apgar score	0.86(0.83-0.89)	<0.0001		1.00		1.14(1.12-1.16)	<0.0001		1.23(1.20-1.25)	<0.0001		1.33(1.29-1.37)	<0.0001		1.47(1.42-1.52)	<0.0001	
Excluding women with eclampsia, gestational hypertension or diabetes																	
Preterm birth	1.36(1.35-1.38)	<0.0001		1.00		0.98(0.97-0.99)	<0.0001		1.05(1.04-1.05)	<0.0001		1.10(1.09-1.11)	<0.0001		1.18(1.16-1.20)	<0.0001	
Low birthweight	1.66(1.64-1.69)	<0.0001		1.00		0.78(0.77-0.79)	<0.0001		0.71(0.70-0.72)	<0.0001		0.66(0.64-0.67)	<0.0001		0.63(0.61-0.64)	<0.0001	
Macrosomia	0.54(0.53-0.55)	<0.0001		1.00		1.52(1.51-1.53)	<0.0001		1.88(1.87-1.89)	<0.0001		2.22(2.19-2.24)	<0.0001		2.66(2.62-2.69)	<0.0001	
SGA	1.57(1.55-1.59)	<0.0001		1.00		0.80(0.79-0.81)	<0.0001		0.76(0.75-0.77)	<0.0001		0.73(0.72-0.74)	<0.0001		0.70(0.69-0.71)	<0.0001	
LGA	0.54(0.53-0.56)	<0.0001		1.00		1.57(1.56-1.58)	<0.0001		1.99(1.97-2.01)	<0.0001		2.39(2.37-2.41)	<0.0001		2.89(2.86-2.92)	<0.0001	
Low Apgar score	0.88(0.85-0.91)	<0.0001		1.00		1.13(1.11-1.15)	<0.0001		1.22(1.20-1.24)	<0.0001		1.34(1.31-1.37)	<0.0001		1.55(1.51-1.59)	<0.0001	
BMI, body mass index; LGA, large for gestational age; SGA, small for gestational age; OR, odds ratio; CI, confidence interval; Ref, reference
ORs (95% CIs) were adjusted for maternal age at delivery, race/ethnicity, education levels, marital status, smoking status during pregnancy, live-birth order, infant sex, gestational age (for low birthweight, macrosomia, or low Apgar score only), and total number of prenatal care visits; maternal pre-pregnancy normal weight was the reference group.
